# Molecular Biomarkers of Radiosensitivity and Radioresistance in Cervical Cancer: A Systematic Review

**DOI:** 10.3390/ijms27157033

**Published:** 2026-08-05

**Authors:** Anamaria Hermina Girbovan, Cristina Balan, Alexandra Timea Kirsch-Mangu, Eva Fischer-Fodor, Patriciu Achimas-Cadariu

**Affiliations:** 1 Department of Radiation Oncology, “Iuliu Hațieganu” University of Medicine and Pharmacy, 400012 Cluj-Napoca, Romania; girbovan.anamaria.hermina@elearn.umfcluj.ro (A.H.G.);; 2 Department of Radiation Oncology, County Hospital Mures, 540072 Târgu Mureș, Romania; 3 Department of Medical Physics, “Prof. Dr. I. Chiricuta” Institute of Oncology, 400015 Cluj-Napoca, Romania; 4 Department of Radiation Oncology, “Prof. Dr. I. Chiricuta” Institute of Oncology, 400015 Cluj-Napoca, Romania; 5 Tumor Biology Department, “Prof. Dr. I. Chiricuta” Institute of Oncology, 400015 Cluj-Napoca, Romania; 6 Department of Surgical Oncology and Gynecologic Oncology, “Iuliu Hațieganu” University of Medicine and Pharmacy, 400012 Cluj-Napoca, Romania; 7 Department of Surgical Oncology, “Prof. Dr. I. Chiricuta” Institute of Oncology, 400015 Cluj-Napoca, Romania

**Keywords:** uterine cervical neoplasms, radioresistance, radiotherapy, biomarkers, tumor, DNA damage response, cancer stem cells, hypoxia, signaling pathways

## Abstract

The primary aim of this review is to summarize current evidence from clinical and pre-clinical studies on endogenous molecular biomarkers associated with radiosensitivity and radioresistance in cervical cancer, including patients treated with photon-based radiotherapy, cervical cancer cell lines, and xenograft models, and to evaluate the association of these biomarkers with radiotherapy response, residual disease, recurrence and survival, and experimental measures of radiosensitivity. A systematic literature review was conducted for studies published over the last 10 years that evaluated associations between genomic, epigenetic, or protein biomarkers and radiotherapy response or survival outcomes in cervical cancer. Eligible studies included in the current analysis summarize clinical, translational, and pre-clinical studies in correlation with photon-based radiotherapy. Research focusing exclusively on non-coding RNAs, exogenous radiosensitizers, or non-photon modalities was excluded. In total, 112 studies were identified, and 46 of them met the inclusion criteria. The identified biomarkers clustered into several key biological processes: DNA damage response and cell cycle regulation, cancer stemness, hypoxia and microenvironment, epigenetic and transcriptional regulation, and signaling pathways, including exosome-mediated communication. Most markers were linked to radioresistance and adverse outcomes, whereas a smaller subset was associated with increased radiosensitivity. A limited group of biomarkers was linked to clinical outcomes such as local control, residual disease, or survival, and emerging multi-marker protein signatures suggested that combinatorial approaches may outperform single-marker strategies. Radiosensitivity in cervical cancer is regulated by a network of biological pathways. Validated, integrated biomarker panels that capture DNA repair proficiency, stemness, hypoxia adaptation, and key signaling pathways are needed to improve risk stratification and enable biomarker-guided radiosensitization.

## 1. Introduction

Cervical cancer (CC) is the fourth most common cancer in women, accounting for 6.5% of newly diagnosed oncologic cases in 2020, with an incidence rate of 3.1% and mortality rate of 3.4% [1]. Estimates suggest that every two minutes, a woman dies from cervical cancer, and globally, approximately 270,000 deaths are recorded each year [2]. The treatment for early-stage cervical cancer consists of either surgery or concurrent chemoradiotherapy (CCRT) followed by image-guided brachytherapy (IGBT), whereas the treatment for locally advanced disease typically involves CCRT followed by IGBT [3]. Even in the modern era of image-guided techniques used in radiotherapy (RT) and brachytherapy, the retroEMBRACE study reports a rate of up to 30% relapses after treatment, out of which 9% were local failures, highlighting the need for reliable biomarkers to predict treatment response earlier during therapy. Moreover, this finding emphasizes the need for research aimed at developing serological, imaging, and pathological biomarkers for the early prediction of treatment response [4]. A biomarker is generally defined as a measurable indicator of normal biological processes, pathogenic processes, or pharmacologic responses to a therapeutic intervention and may encompass genetic, epigenetic, proteomic, or metabolic parameters. The development of combination therapies that increase the tumor sensitivity to radiation could be guided by biomarkers, which serve as a foundation for customizing cancer treatment by an independent signal of radiosensitivity (RS) and radioresistance (RR) [5]. Intrinsic radiosensitivity refers to the inherent variation in how different tumor cell types respond to radiation, which is not attributable to external factors or differences in DNA repair capacity but rather to intrinsic differences in cellular susceptibility to the induction of irreparable damage [6]. Conventional radiotherapy regimens have historically been designed using radiobiological parameters, such as organ-specific α/β ratios derived from the linear–quadratic model. Therefore, patients with biologically heterogeneous tumors receive identical dose prescriptions, thus offering limited opportunities for treatment personalization [7]. Photon-based external-beam radiotherapy and brachytherapy, delivered concurrently with platinum-based chemotherapy, constitute the established global standard of care for cervical cancer and account for the majority of patients treated worldwide [3,8]. Particle therapy, including proton and carbon ion radiotherapy, provides a favorable dose distribution; however, it is limited to a few highly specialized centers due to significant infrastructural, technical, and financial constraints [8]. In CC, its use is currently restricted to selected clinical scenarios, such as reirradiation of recurrent disease, and the available evidence in locally advanced cervical cancer (LACC) is limited to a few phase I/II clinical trials [9,10]. Due to the radiobiology and relative biological effectiveness of particle beams differing from those of photons and clinical evidence for particle therapy in cervical cancer being limited, we deliberately restricted our scope to photon-based modalities to minimize radiobiological heterogeneity [11,12,13]. Candidate molecular biomarkers and gene signatures linked to radiotherapy response in cervical cancer have started to be found in recent translational and bioinformatic studies. These include differentially expressed genes, pathway-based radiosensitivity genes, and chemokines that promote acquired radioresistance [14]. Although several reviews have addressed radioresistance in cervical cancer, most have adopted a narrower or broader scope than the present work. Several reviews have previously examined the molecular basis of radioresistance in cervical cancer. Narrative syntheses have summarized the principal biological mechanisms underlying radiotherapy resistance [15,16]. A substantial body of literature has focused specifically on non-coding RNAs, particularly long non-coding RNAs, as regulators of radiosensitivity [17,18]. Other narrative reviews have focused specifically on exogenous molecules such as polyphenolic compounds, β-Sitosterol, nanoparticles, immunotherapy, biological therapies, triapine, and metformin used as radiosensitizing agents [19,20,21,22,23,24].

Taking this into consideration, we performed a systematic review to synthesize current evidence on endogenous molecular biomarkers of RS and RR in cervical cancer, with the aim of identifying the most promising candidates, together with biological pathways for future translational validation and biomarker-guided personalization of radiotherapy. This review evaluates clinical biomarker data alongside pre-clinical mechanistic findings to enhance the biological plausibility and translational relevance of the identified biomarkers [25]. To our knowledge, no systematic review has specifically synthesized the evidence on endogenous molecular biomarkers of radiosensitivity in photon-based radiotherapy for cervical cancer while integrating both clinical and pre-clinical data. To address this gap, we deliberately restricted our scope to endogenous protein- and gene-level molecular biomarkers, excluding non-coding RNAs and exogenous radiosensitizers, and to photon-based external-beam radiotherapy and brachytherapy to reduce heterogeneity and enhance relevance.

## 2. Methods

### 2.1. Study Design and Protocol

This review was carried out in accordance with the Preferred Reporting Items for Systematic Reviews and Meta-Analyses (PRISMA) guidelines [8]. The inclusion criteria for pertinent studies were established using the Population, Intervention, Comparison, and Outcome (PICO) model. The population includes patients with cervical cancer treated with photon-based radiotherapy for clinical studies, while for pre-clinical studies, cervical cancer cell lines and xenograft models were used to investigate RS and RR. The interventions analyzed were the presence or absence of expression level or alteration of endogenous molecular biomarkers (genomic, epigenetic, and protein markers, including factors related to DNA damage response, stemness, hypoxia, and signaling pathways) measured in human tumors, tissues, or pre-clinical models. The clinical and pre-clinical comparisons included high versus low biomarker expression or radiosensitive versus radioresistant tumors, as well as radiosensitive versus radioresistant cell lines or animal models with or without biomarker manipulation. The outcomes included clinical goals such as radiotherapy response (complete response or residual disease), local or distant recurrence, disease-free survival (DFS), and overall survival (OS). Pre-clinical outcomes included measures such as clonogenic survival following irradiation, DNA repair, apoptosis, and tumor growth in xenograft models.

### 2.2. Information Sources and Search Strategy

The articles were searched using the PubMed database using the following keywords: (“Cervical cancer” OR “Cervical carcinoma”) AND (“Radiosensitivity” OR “Radiosensitizing agents” OR “Radioresistance”) AND (“Biomarkers” OR “Predictive factors” OR “Survival” OR “Response”) AND (“Radiotherapy” OR “Radiation therapy”). In addition, the reference lists of studies and reviews were manually screened. Full-text articles were retrieved when needed.

### 2.3. Inclusion and Exclusion Criteria

The following inclusion and exclusion criteria were used for study selection:

#### 2.3.1. Inclusion Criteria

1. Studies published between January 2015 and December 2025.
2. Studies published in English.
3. Studies that examined the correlation between different biomarkers and radiosensitivity in cervical cancer.
4. Studies that used X-ray irradiation with photons.

#### 2.3.2. Exclusion Criteria

1. Studies published as abstracts, letters to the editor, or book chapters.
2. Systematic reviews and meta-analyses.
3. Studies focusing on other malignancies than cervical cancer.
4. Studies that used other types of irradiation such as protons, carbons, or neutrons.
5. Studies exclusively investigating non-coding RNAs (microRNAs and long non-coding RNAs) as radiosensitivity biomarkers, as these represent a distinct class of regulators that have been extensively reviewed elsewhere and would require a separate methodological framework [18,19].
6. Studies evaluating exogenous drugs or radiosensitizing agents without assessing a specific molecular biomarker radiosensitivity or radioresistance association.
7. Retracted articles were excluded.

### 2.4. Study Selection Process

All recovered records were uploaded into a reference management program, and duplicates were deleted. Titles and abstracts were screened by two independent reviewers (A.H.G. and C.B.). In case of disagreements, a third independent reviewer (E.F.F.) was involved to reach consensus. Afterward, the same reviewers retrieved and assessed the full text of the studies included.

### 2.5. Data Collection Process and Data Items

Data extraction was conducted separately by the two reviewers (A.H.G. and C.B.) utilizing a standardized data collection form. The information extracted consisted of study characteristics (author, year, and study design), the biomarker studied, biological mechanism or pathway involved, number of patients, cell lines used, xenograft models used, and main outcomes. Discrepancies among reviewers were addressed by verifying the article text (E.F.F.).

### 2.6. Risk-of-Bias Assessment

A formal, validated risk-of-bias tool was not applied. However, two reviewers qualitatively appraised key methodological aspects of each clinical and translational study, including study design, patient selection, timing and methods of biomarker assessment, outcome definition, and completeness of reporting, and these considerations were taken into account when interpreting the results. For pre-clinical studies, we descriptively evaluated the use of appropriate controls, irradiation protocols, biomarker manipulation, and outcome assessment. No automation tools were used in this process.

### 2.7. Data Synthesis

The predefined inclusion and exclusion criteria were applied to select the studies eligible for this review. Following screening, the selected studies were categorized for synthesis based on the predominant biological mechanism or biomarker classification such as DNA damage response and cell cycle, cancer stem cell and stemness, hypoxia and microenvironment, epigenetic and transcriptional regulation and signaling pathways, exosomes, and other mechanisms. Due to the marked heterogeneity in biomarker types, assays, and outcome definitions, no statistical pooling or imputation of missing summary statistics was performed. Endpoints such as local recurrence, disease-free survival, overall survival, or effect estimates were extracted and presented as reported by the original authors. The results were synthesized without performing a quantitative meta-analysis. No formal statistical investigations of heterogeneity were conducted because quantitative pooling was not performed due to substantial clinical and methodological heterogeneity across included studies. Sources of heterogeneity such as differences in biomarkers studied, patient populations, and outcome definitions were explored qualitatively by comparing findings within predefined biological domains. The results of individual studies were summarized in structured tables organized by biological mechanism (Table 1, Table 2, Table 3, Table 4 and Table 5). Each table reports for every study the first author and year, biomarker, underlying biological mechanism, type of evidence (clinical, in vitro, or in vivo), study design and sample, the direction of the effect on RS or RR, and a concise conclusion. This tabular format was created to visually organize data within each mechanistic category and to facilitate a synthesis of biomarker and radiosensitivity associations.

## 3. Results

### 3.1. Study Selection and Characteristics

**Figure 1:**
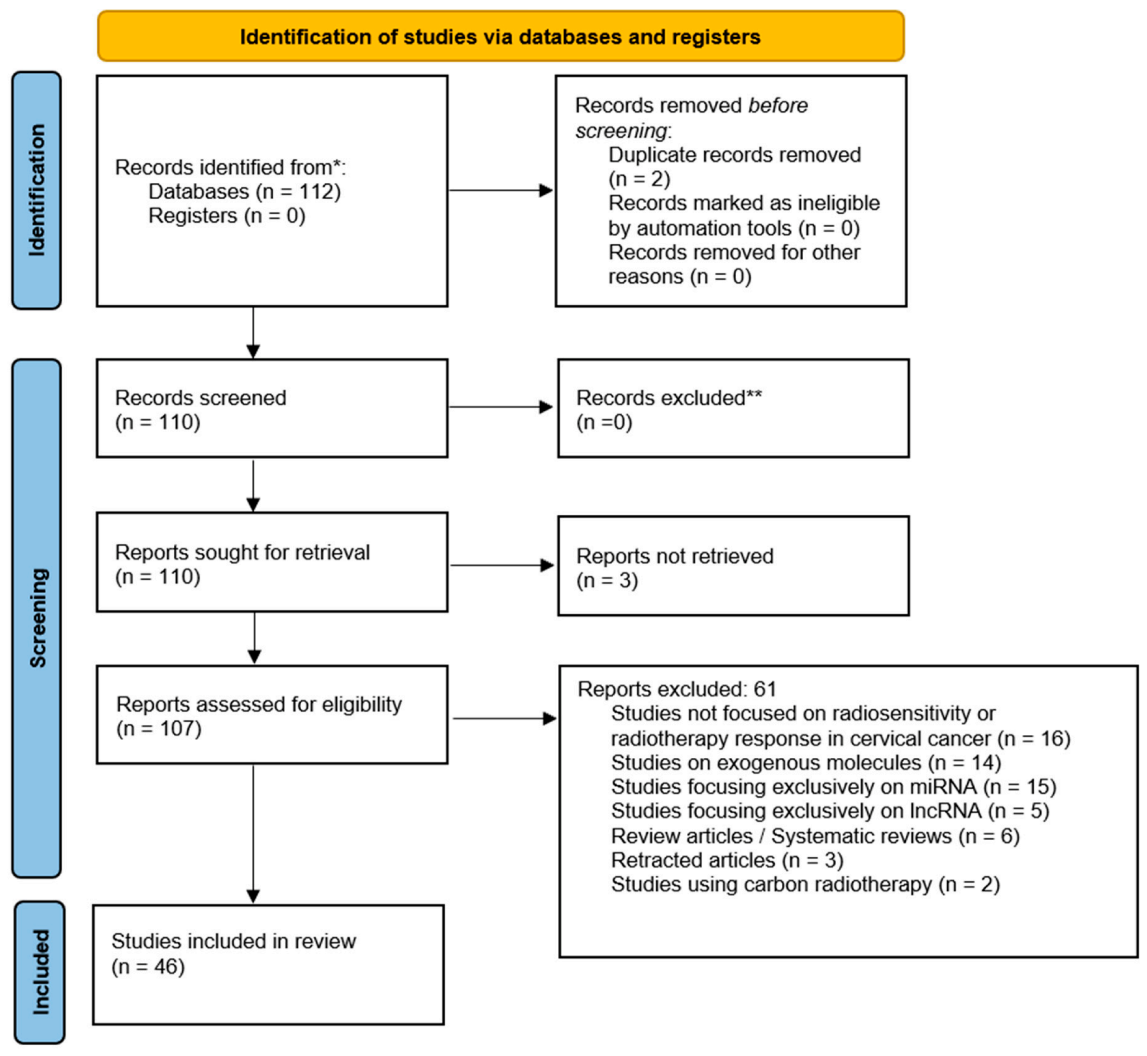
Flow diagram of literature search, screening, and inclusion or exclusion of studies. * Records identified through database searching only (PubMed); no additional registers or other sources were used. ** No records were excluded at the screening stage (title/abstract); all screening was performed manually by the reviewers.

The literature research generated 112 studies. Inclusion and exclusion criteria were applied, and the two duplicates were excluded. A total of 110 studies were included in the initial selection. The records were screened by title and abstract, and all were sought for full-text review. In total, 3 reports could not be retrieved, leaving 107 full-text articles assessed for eligibility. Taking into consideration that the analysis was limited to endogenous molecular biomarkers directly linked to RS, studies not focusing on radiosensitivity or radiotherapy response in cervical cancer, involving exogenous radiosensitizing agents or non-coding RNAs (miRNAs or lncRNAs), review articles, retracted articles, and studies on carbon ion radiotherapy were excluded. A total of 46 studies met the inclusion criteria and were included in the qualitative synthesis (Figure 1).

### 3.2. DNA Damage Response and Cell Cycle-Related Biomarkers

DNA damage is the main mechanism by which ionizing radiation exerts its cytotoxic effect. When DNA damage is detected, it triggers a chain reaction of DNA damage response (DDR) signaling pathways that determine the cell fate: repair of the damage or apoptosis. Therefore, DNA damage response is one of the principal mechanisms of RR development [26].

About a third of the studies focused on DNA damage response and cell cycle-related biomarkers. These studies included both purely pre-clinical work and translational series that combined patient groups with in vitro or in vivo models. *IER5* (Immediate early response 5) was evaluated as a potential radiosensitive marker of CC in a prospective clinical study on 43 patients. *IER5* was induced by RT in a dose-dependent manner and was positively correlated with apoptosis of CC cells while being negatively correlated with tumor dimension. However, *IER5* expression did not differ significantly between the radioresistant and radiosensitive groups, indicating that despite its proposed role in promoting irradiation-induced apoptosis, IER5 levels were not clinically associated with radiosensitivity in this cohort. This finding may be limited by the small sample size [27,28]. Another study conducted by Zheng et al. showed in vitro that suppressing *PAF1* (RNA Polymerase II-Associated Factor 1) reduces IER5, which contributes to radioresistance. This finding supports a potential radiosensitizing role for *IER5* in CC, although this has yet to be confirmed clinically [29]. Another radiosensitivity biomarker is represented by *SETD3* (SET Domain Containing 3), a histone methyltransferase implicated in DNA damage-induced apoptosis, which was examined by a prospective study integrating clinical data with in vitro and in vivo models utilizing the SiHa cell line. *SETD3* expression exhibited an inverse correlation with radioresistance in cervical cancer. Its knockdown reduced irradiation-induced cell death, DNA damage, and apoptosis, whereas its overexpression was sufficient to overcome radioresistance in vivo. *SETD3* increased radiosensitivity by lowering the levels of KLC4 (Kinesin Light Chain 4) and lowering the production of nitric oxide by downregulating iNOS/eNOS (i—inducible; e—endothelial; NOS—nitric oxide synthases). Therefore, *SETD3* could represent a potential therapeutic target to restore radiosensitivity in radioresistant CC tumors [30,31]. Two proteins related to tumor suppressors, RIZ1 (Retinoblastoma-protein interacting zinc-finger gene 1) and USP53 (Ubiquitin-Specific Peptidase 53), were found to modulate radiosensitivity in CC. *RIZ1* overexpression synergistically augmented the anti-tumor efficacy of radiotherapy by increasing DNA damage and apoptosis both in vitro and in vivo. In a retrospective cohort of 40 patients, native *USP53* expression was positively correlated with radioresistance, with radiotherapy response rates being significantly lower among *USP53*-positive patients. Conversely, *USP53* knockdown compromised DDB2 (Damage-Specific DNA Binding Protein 2)-mediated DNA repair capacity and disrupted cell cycle progression through dysregulation of the *CDK1/CHK2* (Cyclin-Dependent Kinase 1 and Checkpoint Kinase 2) pathway, thereby sensitizing tumor cells to radiation and negatively affecting cell survival post-irradiation. These findings collectively demonstrate *RIZ1* as a radiosensitizer and *USP53* as a driver of radioresistance whose inhibition restores radiosensitivity in cervical cancer [32,33,34,35].

Among the DNA damage response and cell cycle-related biomarkers associated with radioresistance, several proteins were shown to promote resistance by enhancing DNA repair capacity and survival signaling. Fu et al. showed that *SKP2* (S-phase kinase-associated protein 2) overexpression was correlated with locoregional recurrence and poor prognosis in a retrospective study cohort of 149 patients; furthermore, in vitro, *SKP2* stimulated double-strand break repair [36,37]. Similarly, *NEK2* (Never in Mitosis (NIMA)-related kinase 2) overexpression was associated with advanced staging and lymph node metastasis in a retrospective cohort of 41 patients, while its knockdown sensitized cervical cancer cells to radiation through potentiation of DNA damage and suppression of DNA repair mechanisms. Furthermore, *NEK2* activated Wnt1/β-catenin signaling, which further promotes radioresistance, making it a potential therapeutic target in CC [38,39]. The *HNF1α/YTHDF3/RAD51D* (Hepatocyte Nuclear Factor 1 alpha/YTH Domain Family Protein 3/RAD51 Paralog D) axis was upregulated in radioresistant tissues in a prospective study of 20 patients and in vitro CC cells, while on the other hand, its disruption reduced radioresistance both in vitro and in vivo. Mechanistically, *HNF1α* transcriptionally activated the m6A reader *YTHDF3*, which subsequently promotes the translation of the homologous recombination factor *RAD51D*, thereby creating an *HNF1α/YTHDF3/RAD51D* signaling axis that regulates radioresistance in vitro and in murine models. These data demonstrate that *HNF1α*-mediated mRNA stability regulation of *RAD51D* is a principal factor in radioresistance and a potential therapeutical target in CC [40].

Furthermore, another study on SiHa and CaSki cell lines, validated in vitro on murine models, showed that *RhoC* (Ras homolog gene family, member C) controls a pro-repair transcriptional program in cells that are resistant to radiation by increasing the levels of several DNA repair components, such as *RAD50* ((*RAD50* Double-Strand Break Repair Protein), *BRCA2* (Breast Cancer gene 2), *NBS1* (Nijmegen Breakage Syndrome 1), *MRE11* (Meiotic Recombination 11 homolog), and pH2AX (phosphorylated H2AX/gamma-H2AX). High *ROCK2* (Rho-associated Coiled-coil-containing protein Kinase 2) activity further supported this phenotype by interacting with the DNA repair system, promoting cell survival, and facilitating cell cycle progression. Inhibition of *RhoC* and *ROCK2* through pharmacologic or siRNA methods consistently enhanced radiosensitivity, suggesting that the *RhoC/ROCK2* axis could be targeted in CC in order to promote radiosensitivity [41]. Furthermore, in an experimental in vitro-only study, *SND1* (Staphylococcal Nuclease Domain-containing protein 1) was shown to promote resistance via *ATM* (Ataxia Telangiectasia Mutated) pathway activation and G2/M checkpoint maintenance, while its silencing significantly enhanced radiosensitivity [42].

Another in vitro study on HeLa and other cellular models demonstrated that *POT1* (Protection Of Telomeres 1), a critical component of telomere protection and the DNA damage response, contributes to the radioresistance of CC cells. The depletion of *POT1* increased radiosensitivity by impairing the repair of telomeric DNA damage induced by radiation, hence diminishing genomic stability and altering the telomerase localization at telomeres. The simultaneous inhibition of both *POT1* and telomerase resulted in a more pronounced impact. The results suggest that *POT1* maintenance is essential for radioresistance [43,44].

Concerning the *EGFR/ERCC1/*p53 (Epidermal Growth Factor Receptor)/(Excision Repair Cross-Complementation group 1)/(Protein 53) axis, it was shown that radiation-induced upregulation of *ERCC1* and p53 was observed only in tumor tissues, not in cell lines, highlighting that in vitro results do not always replicate the intricate cellular and molecular interactions occurring in clinical cohorts [45].

Finally, survivin, a member of the inhibitor of apoptosis (IAP) family, has been demonstrated in in vitro and in vivo models to function as a crucial mediator of radioresistance in cervical cancer. In C33A cells, the knockdown of survivin via synthetic siRNA markedly increased radiosensitivity after 6 Gy irradiation by elevating DNA damage, genomic instability, apoptosis, and G2/M arrest, concomitant with the upregulation of the pro-apoptotic factor *PUMA* (p53 Upregulated Modulator of Apoptosis). On the other hand, the inhibition of *PUMA* reversed this radiosensitizing effect. In vivo, reducing survivin similarly enhanced tumor response to irradiation, suggesting that survivin could be a promising therapeutic target for radiosensitization [46].

In summary, DDR and cell cycle checkpoints have emerged as important factors in radioresistance in cervical cancer, with converging data from these studies indicating several potentially therapeutic targets that may be utilized to enhance radiosensitivity (Table 1).

**Table 1:** Summary of studies concerning DNA damage response and cell cycle-related biomarkers.

First Author (Year)	Biomarker	Biological Mechanism	Type of Evidence	Study Type/Sample	Effect Direction	Conclusion
de Almeida VH et al. (2018) [45]	*EGFR*, *ERCC1*, and p53	Cell division/survival signaling and DNA repair	Clinical In vitro	Prospective, n = 10; experimental CaSki * and C33A *	NA	Radiation induced at least one of *EGFR/ERCC1*/p53 in tumors but not cell lines
Zhou J et al. (2020) [46]	Survivin	Inhibitor apoptosis (IAP) protein family	In vitro In vivo	Experimental C33A * + siRNA; murine models	↑ RR (when expressed); ↑ RS when inhibited	Targeting survivin enhanced radiosensitivity
Fu X et al. (2024) [42]	*SND1*	DNA damage response/ATM	In vitro	HeLa *, CaSki *	↑ RR	*SND1* promotes RR; silencing it promotes RS
Li Q et al. (2023) [44]	*POT1*/telomerase	Telomere protection/DNA repair	In vitro	HeLa *, 293T *, MRC5 *, and U2OS * + siRNA	↑ RR	*POT1* is a radiosensitization target
Xu T et al. (2020) [39]	*NEK2*	Mitosis/Wnt-beta-catenin	Clinical In vitro In vivo	Retrospective, n = 41; HeLa * and SiHa * + siRNA; murine models	↑ RR	*NEK2*-driven Wnt1/β-catenin pathway activation promotes oncogenesis and RR in CC
Li Q et al. (2019) [30]	*SETD3*	DNA damage/apoptosis	Clinical In vitro In vivo	Prospective, SiHa * and SiHa-XR * + siRNA; animal models	↑ RS	*SETD3* enhances RS in cervical cancer by suppressing *KLC4* expression and reducing NO generation via iNOS/eNOS downregulation
Du H et al. (2023) [40]	*HNF1α/YTHDF3/RAD51D*	m6A regulation/homologous recombination	Clinical In vitro In vivo	N = 20 (10 RS, 10 RR); HeLa *, ME180 *, SiHa *, and MS751 *; murine models	↑ RR	HNF1α upregulation drives RR in CC through the *HNF1α/YTHDF3/RAD51D* regulatory axis
Pranatharthi A et al. (2019) [41]	*RhoC/ROCK2*	Cytoskeleton/DNA repair	In vitro In vivo	SiHa * and CaSki * + siRNA; murine models	↑ RR	*RhoC* and *ROCK2* inhibition increases the radiosensitivity of cancer cells
Yang S et al. (2018) [32]	*RIZ1*	Tumor suppressor/apoptosis/DNA damage	In vitro In vivo	HeLa * and SiHa * + overexpression plasmid; murine models	↑ RS (when overexpressed)	*RIZ1* overexpression combined with RT enhanced apoptosis and DNA damage
Zhou Q et al. (2022) [35]	*USP53*	*CDK1/CHK2/DDB2*/DNA repair	Clinical In vitro	Retrospective, n = 40; SiHa * + siRNA	↑ RR (when expressed); ↑ RS when inhibited	*USP53* promotes radioresistance via *DDB2*-mediated DNA repair; its knockdown induces G2/M cell cycle arrest and restores radiosensitivity
Fu HC et al. (2017) [36]	*SKP2*	Cell cycle/proliferation/survival	Clinical In vitro	Retrospective, 149 patients; HeLa * and C33A * + siRNA	↑ RR	*SKP2* predicts recurrence and poor RT response
Liu Y et al. (2017) [27]	*IER5*	DNA damage response and apoptosis	Clinical	Prospective, n = 43	↑ RS	RT-induced *IER5* → ↑ apoptosis; inversely correlated with tumor size

Abbreviations: *, cell lines (human cell models); CC, cervical cancer; in vitro, cell line studies; in vivo, animal model studies; RR, radioresistance; RS, radiosensitivity; RT, radiotherapy.

### 3.3. Cancer Stem Cell (CSC) and Stemness-Associated Biomarkers

Within the cancer stem cell and stemness-associated category, *SOX2* (SRY-Box Transcription Factor 2) was the most frequently reported marker, cited in three studies and generally linked to an adverse clinical phenotype. However, the evidence was not entirely consistent across all cohorts. In a prospective study involving 56 patients led by Huang et al., elevated *SOX2* expression correlated with advanced International Federation of Gynecology and Obstetrics (FIGO) stage (37.84% vs. 10.53%, *p* = 0.032), increased the local recurrence (32.4% vs. 10.5%, *p* = 0.005) and cancer-related mortality (46.0% vs. 15.8%, *p* = 0.026), significantly diminished survival (median OS 28.9 vs. 60.3 months, *p* < 0.01), and a reduced local-recurrence-free interval (31.3 vs. 92.2 months, *p* < 0.05). Additionally, *SOX2* knockdown in radioresistant HeLa and SiHa cell models reinstated radiosensitivity in vivo [47]. In a prospective series of 59 patients, Chakrabarti D et al. observed a similar trend, with high *SOX2* tumors exhibiting reduced 3-year overall survival (53% vs. 60%, *p* = 0.856) and disease-free survival (50% vs. 65%, *p* = 0.259) in comparison to low *SOX2*. However, these differences did not reach statistical significance [48]. Similarly, in a larger retrospective cohort of 139 patients, *SOX2* alone showed a tendency to negatively impact OS and DFS without significantly differentiating 5-year OS (54.3% vs. 60.0%, *p* = 0.598) and 5-year DFS (48.4% vs. 64.4%, *p* = 0.141). On the other hand, high SOX2 expression in association with low P16INK4A, a pivotal tumor suppressor protein that modulates the cell cycle by inhibiting G1/S-phase transition of the cell cycle, was identified in a subgroup with notably poor 5-year disease-free survival (26.8% vs. 70.2%, *p* = 0.009), indicating that the prognostic significance of *SOX2* may be contingent upon its interaction with other cancer stem cell and cell cycle markers [49,50]. Furthermore, ALDH (Aldehyde dehydrogenase) was identified as another biomarker correlated with radioresistance in a retrospective clinical study. A cut-off value of ALDH score was associated with a 3-fold increase in the risk of incomplete response to radiotherapy (OR 3.127, 95% CI 1.034–9.456, *p* = 0.043) [51]. Moreover, *Nurr1* (Nuclear Receptor-Related 1 Protein), a nuclear protein receptor transcription factor, has also been identified as a major HPV-related driver of radioresistance within this CSC/stemness axis. In vitro and in vivo studies revealed that *Nurr1* promoted self-renewal, tumorigenicity, and radioresistance by co-activating the MEK/ERK (Mitogen-activated protein kinase Kinase/Extracellular signal-Regulated Kinase) and PI3K/AKT/mTOR pathways (Phosphoinositide 3-Kinase/Protein Kinase B/mechanistic Target of Rapamycin), with AKT signaling being essential for *Nurr1*-enhanced resistance to irradiation. Nurr1 was upregulated in primary cervical cancer spheroids and induced by HPV E6 [52,53]. Nonetheless, evidence for *Nurr1* remains limited to in vitro and in vivo models, and clinical studies are needed to confirm whether this relationship with radioresistance holds in patient cohorts. Lastly, CD71, a transferrin receptor implicated in extracellular iron acquisition, also functioned as a marker indicative of HPV-driven cancer stem cell-like characteristics associated with radioresistance. In a tissue microarray series of 140 CC, CD71 expression was elevated in tumors compared to normal tissue. It was particularly concentrated in HPV-positive cell lines, where CD71^+^ cells exhibited superior post-irradiation clonogenic survival, accelerated self-renewal, and increased tumorigenicity relative to CD71^−^ cells. Furthermore, the HPV E6 oncoprotein expands the CD71^+^ subpopulation, whereas CD71 knockout in HPV-positive SiHa cells, especially when coupled with CD55 inhibition, significantly enhances radiosensitivity. While these results support a mechanistic link between CD71 and radioresistance, clinical evidence is presently restricted to CD71 overexpression in tumor versus normal tissue, and studies correlating CD71 with radiotherapy outcomes in patient cohorts are still needed [54].

In summary, among the cancer stem cell and stemness-associated biomarkers, *SOX2* emerged as the most clinically validated marker, though its clinical evidence was not uniformly significant; ALDH1 showed a significant association with RT response but requires validation against other clinical endpoints, while *Nurr1* and CD71 are supported only by pre-clinical data (Table 2).

**Table 2:** Summary of studies concerning CSC and stemness-associated biomarkers.

First Author (Year)	Biomarker	Biological Mechanism	Type of Evidence	Study Type/Sample	Effect Direction	Conclusion
Huang C et al. (2018) [47]	*SOX2*	Tumor growth, metastasis, and drug resistance	Clinical In vitro In vivo	Prospective, N = 56; HeLa/SiHa * and RR variants; murine models	↑ RR	*SOX2* high → worse OS + LR; suppression restores RS
Chakrabarti D et al. (2024) [48]	*SOX2/OCT4*	Stemness/embryonic pathways	Clinical	Prospective N = 59	↑ RR	High *SOX2* and low *OCT4* showed a non-significant trend toward worse 3-year OS and DFS
Fahmi MN et al. (2023) [51]	ALDH1	Stemness/oxidative stress	Clinical	Retrospective, n = 58	↑ RR	ALDH score ≥ 166.05 → 3× risk of incomplete RT response
Wan PK et al. (2021) [52]	*Nurr1*	MEK/ERK, PI3K/Akt/mTOR and self-renewal	In vitro In vivo	C33A *, CaSki *, SiHa *, and C4-1 * +siRNA; murine models	↑ RR	HPV E6 and *Nurr1* co-activate MEK/ERK and PI3K/Akt/mTOR signaling to promote proliferation, migration, invasion, and self-renewal
Leung TH et al. (2019) [54]	CD71	HPV E6/iron uptake/stem-like phenotype	Clinical In vitro In vivo	TMA IHC series (140 cervical cancer vs. 10 normal tissues); C33A *, C4-1 *, CaSki *, and SiHa + siRNA; BALB/c nude	↑ RR	HPV-E6 enriches CD71+ population → RR; CD71 + CD55 knockdown → ↑ RS
Fu HC et al. (2018) [49]	P16INK4A/*SOX2*/ALDH1A1	Cell cycle + cancer stem cell markers	Clinical In vitro	Retrospective, n = 139; HeLa * + shRNA P16INK4A	↑ RR (low P16INK4A)	Low P16INK4A + high ALDH1/*SOX2* → tendency to worsen 5y OS/DFS

Abbreviations: *, cell lines (human cell models); DFS, disease-free survival; in vitro, cell line studies; in vivo, animal model studies; RR, radioresistance; RT, radiotherapy; TMA, tissue microarray; OS, overall survival.

### 3.4. Hypoxia and Microenvironment-Related Biomarkers

Several hypoxia and microenvironment-related biomarkers were consistently associated with radioresistance in CC (Table 3). For example, ALDH1 emerged as a crucial stemness-associated mediator of radioresistance. In in vitro models using SiHa and HeLa cells, hypoxia treatment promoted a fibroblast-like morphology, enhanced cellular proliferation and cell cycle modifications, and significantly augmented sphere-forming capacity, aligning with the enrichment of a cancer stem cell-like phenotype. In radioresistant cells, hypoxia elevated ALDH1 and the DNA damage checkpoint kinase 2 (*CHK2*). Although hypoxia did not enhance tumor engraftment in vivo, it promoted the formation of larger tumors. These data suggest that hypoxia promotes the development of an ALDH1-positive, stem-like, radioresistant phenotype in CC. These subregions may require dose-escalated RT in order to achieve adequate control [55].

Furthermore, in a prospective study of 149 FIGO IIB–IIIB cervical cancer patients, high IGF-1Rβ (Type 1 Insulin-like Growth Factor Receptor) expression by immunohistochemistry was associated with poorer overall survival (*p* = 0.007). At the same time, GLUT1 (Glucose transporter 1) overexpression showed a trend towards reduced overall survival (*p* = 0.05) [56]. Several in vitro studies showed that chemokines and extracellular matrix (ECM) components shape a radioresistant microenvironment: *CXCL12* C-X-C motif chemokine ligand 12) and *CXCL8* (C-X-C motif chemokine ligand 8/interleukin 8) signaling, as well as the matricellular protein periostin (*POSTN*), promoted survival after irradiation, whereas their inhibition restored radiosensitivity in HeLa- and SiHa-derived models [57,58,59,60]. Moreover, *POSTN* was identified in an exploratory TCGA (The Cancer Genome Atlas) cohort (n = 92, using mRNA) and validated on a retrospective clinical cohort (n = 153, using IHC), where high expression predicted incomplete radiotherapy response (*p* = 0.028) and worse OS (HR 4.005, *p* < 0.001). Furthermore, an exploratory in silico drug sensitivity analysis further suggested that high *POSTN* expression may be linked to resistance to several agents such as Lapatinib, Onalespib, Bromopyruvic acid, Allopurinol, Hypothemycin, the product of Debio 0932, Gefitinib, and Parthenolide [60]. Regarding *CXCL8*, immunohistochemical analysis of a retrospective cohort of 12 cervical cancer patients and paired adjacent normal tissues confirmed that CXCL8 expression was higher in tumor tissue, consistent with GEPIA2 (Gene Expression Profiling Interactive Analysis 2) bioinformatics findings [58]. Finally, Liu et al. conducted an in vitro study on HeLa cervical cancer cells irradiated with 4 Gy, showing that irradiation upregulated *UCP2* (Uncoupling Protein 2), whereas siRNA-mediated *UCP2* inhibition increased ROS, apoptosis, G2/M arrest, and DNA damage, thereby enhancing radiosensitivity. On the basis of these pre-clinical findings, the authors proposed *UCP2* as a potential mitochondrial target for radiosensitization in cervical cancer, although this remains to be validated in clinical cohorts, while *UCP2* emerged as a promising mitochondrial target for novel radiosensitizers in CC [61].

**Table 3:** Summary of hypoxia and microenvironment-related biomarkers.

First Author (Year)	Biomarker	Biological Mechanism	Type of Evidence	Study Type/Sample	Effect on RS	Conclusion
Moreno-Acosta P et al. (2017) [56]	IGF-1R α/β, HIF-1α, Survivin, GLUT1, and CAIX	Hypoxia response	Clinical	Prospective, n = 149	↑ RR	IGF-1R β high expression correlated with worse OS; GLUT1 overexpression marginal association with OS
Fu Z et al. (2018) [57]	*CXCL12*/CD44/CXCR4	Chemokine/tumor microenvironment	In vitro	HeLa * + siRNA CXCL12	↑ RR	*CXCL12* inhibition reduced CD44 and increased apoptosis after irradiation
Huang CQ et al. (2025) [60]	*POSTN*	ECM/tumor microenvironment	Clinical In vitro	92 TCGA + 153 local; HeLa * + siRNA	↑ RR	*POSTN* high → ↓ RT efficacy, worse OS; knockdown → ↑ RS
Hu Q et al. (2025) [58]	*CXCL8* (IL-8)	Chemokine/inflammation	Clinical In vitro	Training cohort TCGA (291) + local (12); HeLa-RR * and SiHa-RR *	↑ RR	*CXCL8* knockdown → restored RS; exogenous CXCL8 → RR in parental CL
Liu CH et al. (2021) [61]	*UCP2*	ROS/cell metabolism/mitochondria	In vitro	HeLa * + UCP2 siRNA	↑ RR	*UCP2* loss increased ROS and enhanced RT-induced apoptosis
Yao T et al. (2020) [55]	Hypoxia/ALDH1	Hypoxia, oxidative stress, and CSC	In vitro In vivo	HeLa * and SiHa RR cell lines; murine models	↑ RR	Hypoxia → ↑ ALDH1, ↑ CHK2 → ↑ RR in RR cell lines

Abbreviations: *, cell lines (human cell models); CL, cell lines; RR, radioresistance; RS, radiosensitivity; RT, radiotherapy; OS, overall survival.

### 3.5. Epigenetic and Transcriptional Regulation

Epigenetic and transcriptional modifications have been identified in multiple studies as contributing to radioresistance (Table 4). Epigenetics describes changes in gene expression without alterations in DNA sequences. The main epigenetic mechanisms include DNA methylation, histone modification, non-coding RNAs, and chromatin remodeling [62]. *PAX1* (Paired Box 1) and *ZNF582* (Zinc Finger Protein 582) promoter methylation were validated by both clinical and in vitro studies as measurable markers of radiotherapy response [63,64]. In a prospective cohort of 125 patients treated with chemoradiotherapy, *PAX1* hypomethylation was correlated with residual disease at 3 months after treatment (multivariate OR 4.433, 95% CI 1.131–17.380, * *p* = 0.033), and a nomogram combining *PAX1* methylation status with tumor size, lymph node metastasis, and pathological type predicted residual tumor with an AUC of 0.823 (95% CI 0.736–0.910). At the molecular level, PAX1 protein overexpression increased the survival of irradiated HeLa and SiHa cells, indicating that reduced promoter methylation and the resulting higher PAX1 expression contributes to radioresistance [65]. On the contrary, in another multicenter prospective study comprising two independent cohorts of LACC (n = 50 each) patients treated with CCRT, higher baseline *ZNF582* promoter methylation (M-index) was significantly associated with RT response (M-index 2356 ± 1985 in responders vs. 641 ± 825 in stable disease, *p* < 0.001; ROC AUC 81.01%, *p* = 0.0002). Furthermore, a transient rise in methylation during RT predicted greater radiosensitivity in the *ZNF582*m-low subgroup. This is mainly because promoter methylation silences *ZNF582* transcription; therefore, high methylation corresponds to low ZNF582 protein, whereas unmethylated tumors retain high protein levels. Accordingly, in vitro ZNF582 protein overexpression induced S-phase arrest via p27/p21/CDK2/4 (Cyclin-dependent kinase inhibitor 1B, p27^Kip1)/p21 (Cyclin-dependent kinase inhibitor 1A, p21^Cip1/WAF1)/CDK2 (Cyclin-dependent kinase 2)/CDK4 (Cyclin-dependent kinase 4) signaling, thereby conferring radioresistance in HeLa cells. Thus, the seemingly opposite associations of methylation and protein expression with radiosensitivity are actually mechanistically consistent, as they reflect the inverse relationship between promoter methylation and protein expression. [66]. Furthermore, *SEPT9* (septine 9) promoter hypermethylation was proposed as an early diagnostic marker; however, *SEPT9* is overexpressed in cervical tumors, where it promotes proliferation, macrophage polarization, and radioresistance through interaction with *HMGB1* and miR-375-regulated tumor-associated macrophage [67].

At the chromatin level, the cancer–testis antigen *GAGE*, particularly the *GAGE12* isoform, was identified as a mediator of radioresistance [68,69]. Nin et al. demonstrated in a retrospective clinical study on 43 patients treated with CCRT followed by BT that higher *GAGE* expression was significantly associated with RR, defined as local and/or distant recurrence within two years after treatment (resistant n = 20 versus sensitive n = 23; mRNA *p* = 0.0028; immunohistochemistry (IHC) intensity *p* = 0.026). The findings in vitro and in vivo showed that GAGE12 localizes to chromatin in a synemin-dependent manner, promotes the association of *HDAC1/2* (Histone deacetylase 1)/(Histone deacetylase 2) with actin, and increases H3K56 acetylation, therefore enhancing chromatin accessibility and DNA repair. Furthermore, *GAGE* knockdown restored radiosensitivity in HeLa/SiHa cells, and high *GAGE* expression in pretreatment biopsies predicted residual tumor after RCT [69]. In parallel, Li et al. demonstrated in vitro that the transcription factor HMGB3 (High Mobility Group Box 3) binds the *hTERT* promoter and upregulates *hTERT* (human Telomerase Reverse Transcriptase) transcription in radioresistant HeLa-RR and SiHa-RR cells, as *HMGB3/hTERT* overexpression supports DNA damage repair and survival after irradiation, whereas *HMGB3* knockdown increased radiosensitivity in vitro and in xenografts. Clinically, high *HMGB3/hTERT* expression in patients was associated with poor response to radiotherapy (local control, complete responders and partial responders versus non-responders: *HMGB3 p* = 0.040, *hTERT p* = 0.049). Furthermore, in a separate tissue microarray of 119 patients, *HMGB3/hTERT* expression was associated with higher TNM (Tumor, Node, and Metastasis) stage and shorter survival, with *HMGB3* identified as an independent prognostic factor (HR 23.48, 95% CI 3.21–171.94, * *p* = 0.0019). These findings support that targeting *HMGB3/hTERT* could represent a potential therapeutic strategy in CC [70].

Transcriptional pausing was identified to contribute to RT response modulation. Zheng et al. demonstrated that *PAF1* (a component of the RNA polymerase II-associated factor 1 complex) overexpression together with low *IER5* expression, assessed by immunohistochemistry and Western blot, was observed in 15 cervical cancer tissues but not in 15 healthy cervical tissues [71]. Moreover, in vitro, it was shown that *PAF1* represses the early immediate response gene *IER5* by binding to downstream enhancers and maintaining RNA polymerase II pausing at the promoter region. Furthermore, *PAF1* knockdown upregulated *IER5* and significantly increased the radiosensitivity of HeLa and SiHa cells [29]. Finally, at a more integrative level, Choi et al. developed a proteomic prediction model in a prospective cohort of 181 patients with LACC treated with CCRT, using reverse-phase protein arrays (RPPAs) to profile 22 candidate proteins in pretreatment frozen tumor samples. A five-protein marker panel consisting of *ERCC1*, CD133 (Cluster of Differentiation 133), HER2 (Human Epidermal Growth Factor Receptor 2), BCL2 (B-cell lymphoma 2), and CAIX (Carbonic Anhydrase IX) independently stratified patients by survival, and the derived risk score was an independent predictor of both progression-free survival (HR 1.6, 95% CI 1.4–1.9, *p* < 0.001) and OS (HR 2.1, 95% CI 1.7–2.5, *p* < 0.001). Combined with two clinical variables (age and FIGO stage), this model was internally validated in a test set and externally explored in the TCGA dataset, where it showed a tendency toward improved OS prediction only in the TCGA cohort, with limited validation likely due to RNA-based data and a small sample size [72]. Collectively, these findings show that epigenetic and transcriptional modifications influence RS in CC at both the mechanistic and the clinical level (Table 4).

**Table 4:** Summary of epigenetic and transcriptional regulation biomarkers.

First Author (Year)	Biomarker	Biological Mechanism	Type of Evidence	Study Type/Sample	Effect Direction	Findings
Li X et al. (2023) [65]	*PAX1* hypomethylation	Tumor suppressor gene/methylation	Clinical In vitro	Prospective, n = 125; HeLa * and SiHa *	↑ RR	*PAX1* hypomethylation associated with residual disease after treatment; increased PAX1 linked to RR
Li Z et al. (2020) [70]	*HMGB3/hTERT*	DNA binding/telomerase activation	Clinical In vitro In vivo	Prospective n = 53; HeLa–RR * and SiHa–RR * + *HMGB3* siRNAs; murine models	↑ RR	*HMGB3* linked to poor RT response; silencing sensitized tumors
Wu N et al. (2022) [66]	*ZNF582* methylation	Cell cycle arrest/promoter methylation	Clinical In vitro	Prospective, n = 50; (cohort 1), n = 50 (cohort 2) Hela *	↑ RS (methylated)	Higher promoter methylation (→ lower protein) predicted better RT response; protein overexpression (in unmethylated tumors) promoted radioresistance via S-phase arrest in vitro
Zheng JJ et al. (2020) [29]	*PAF1/IER5*	RNA Pol II pausing/transcriptional regulation	In vitro	HeLa *, SiHa * + siRNA *PAF1*, siRNA *IER5*, and 15 CC vs. 15 normal tissues (expression only)	↑ RR	*PAF1* contributes to radioresistance through IER5 suppression
Choi CH et al. (2020) [73]	*ERCC1*/CD133/HER2/BCL2/CAIX	Multi-marker RPPA panel	Clinical	Prospective, 181 patients, internal (train/test) + external (TCGA) validation	↑ RR (panel high)	5-marker RPPA panel predicts CCRT response; RS score defined
Jiao X et al. (2019) [67]	*SEPT9* hypermethylation	Epigenetic/macrophage polarization	Clinical In vitro In vivo	Tumor-versus-normal tissue expression (n = 80 paired SS), n = 80; HeLa* and CaSki * + siRNA-*SEPT9* siRNA-*HMGB1*; murine models	↑ RR (OE)	*SEPT9* overexpression → RR; hypermethylation = diagnostic biomarker
Nin DS et al. (2021) [69]	*GAGE/GAGE12*	Chromatin accessibility/HDAC	Clinical In vitro In vivo	Retrospective, n = 43 ; HeLa * and SiHa *; murine models	↑ RR	*GAGE12* inhibits HDAC1/2 → ↑ chromatin access → ↑ DNA repair → RR

Abbreviations: *, cell lines (human cell models); OE, overexpression; RR, radioresistance; RS, radiosensitivity; RT, radiotherapy; SS, surgical specimens.

### 3.6. Signaling Pathways, Exosomes and Other Mechanisms

Various signaling-related biomarkers were linked to intrinsic or acquired radioresistance in cervical cancer (Table 5). Biomarkers can be further classified into different pathways: canonical oncogenic, non-canonical oncogenic, and vesicle mediated. Several studies identified radioresistant molecular alterations mapped to canonical pathways such as *EGFR*, *KRAS/SMAD4*, *PIK3CA*, *mTOR/Bcl-2*, and *TRIP4/hTERT* [74,75,76,77,78]. However, *EGFR* and *PIK3CA* were identified in small clinical series, while *EGFR/PI3K/mTOR/Bcl-2* were demonstrated in vitro in the HeLa cell line and thus require further validation. Non-canonical pathways included *IRAK1*, *FOXM1*, HSP90, CD146, *TIAM2S*, *MICAL2*, and *KLK5* [79,80,81,82,83,84,85].

Proteases and chaperones such as *KLK5* (Kallikrein-Related Peptidase 5) and HSP90 (Heat Shock Protein 90) promoted a radioresistant phenotype by enhancing proliferation, invasion, and survival signaling and by inhibiting irradiation-induced apoptosis and G2/M arrest. In contrast, pharmacologic or genetic inhibition of *HSP90* restored radiosensitivity via CD147 degradation. *KLK5* was clinically validated in a retrospective cohort of 19 patients with CC classified into radiosensitive (13, no recurrence or metastasis within 3 years) and RR (6, recurrence or metastasis ≤ 3 years). *KLK5* expression was significantly higher in the RR group, thus correlating with poorer RT response [85]. Furthermore, Song Q et al. developed radioresistant CaSki and SiHa CC lines by exposure to a cumulative dose of 60 Gy and validated their radioresistance in xenograft models. HSP90 (HSP90α and HSP90β) interacted with *FBXO6* and CD147 in these cells and suppressed *FBXO6*-mediated polyubiquitination and proteasomal degradation of CD147, thereby stabilizing CD147 and promoting radioresistance. Pharmacologic inhibition of *HSP90* induced CD147 ubiquitination and degradation, sensitized radioresistant cervical cancer cells to irradiation in the 2–8 Gy range, and uncovered the *HSP90–FBXO6–CD147* axis as a potential therapeutic target for overcoming radioresistance [81]. Oncogenic pathway components, including *MICAL2*, *EGFR*, *PIK3CA*, *TRIP4*, and *KRAS/SMAD4*, were repeatedly linked to poor radiotherapy response in clinical cohorts and experimental models, mainly through activation of *MAPK* (Mitogen-Activated Protein Kinase), *PI3K/AKT/mTOR*, and RAS-related signaling. Teng et al. demonstrated the association between *MICAL 2* (microtubule-associated monooxygenase, calponin, and LIM domain containing 2) with RR in a prospective cohort of 62 CC patients and in multiple CC cell lines and xenograft models. *MICAL 2* expression is upregulated in tumor tissues and cell lines, where it promotes proliferation, survival, migration, and invasion by activating MAPK and PI3K/AKT signaling and driving EMT. From a functional perspective, *MICAL2* reduced irradiation-induced DNA damage and decreased RS in vitro, whereas *MICAL2* knockdown impaired tumor growth and enhanced RS in mouse models. Furthermore, in a prospective cohort of 62 patients, high MICAL2 expression assessed by IHC correlated with poor response to radiotherapy (local control complete response 39.1% vs. 74.4% in low MICAL2 expression, *p* = 0.006) and decreased DFS (*p* = 0.032) but not with OS [84]. High IHC EGFR (Epidermal Growth Factor Receptor) expression was associated with incomplete response to radiotherapy; 94.1% of *EGFR*-positive tumors showed an incomplete response versus only 5.9% of EGFR-negative tumors (*p* = 0.0001) in a retrospective study of 32 patients with stage IIB-IVB adenocarcinoma CC. These findings indicate that *EGFR* overexpression is correlated with RR in this histological subtype [74]. Complementary data in a different histological setting were reported by de Almeida et al. in a small prospective series of 10 patients with squamous CC, showing upregulation of *EGFR, ERCC1*, and p53 expression in tumor tissues after RT [45] Furthermore, in the wider context of signaling-driven radioresistance, Che et al. identified the transcriptional activator *TRIP4* (Thyroid Hormone Receptor Interactor 4) as overexpressed in CC tissues compared with adjacent normal tissue (n = 15). Functionally, in vitro findings showed that *TRIP4* overexpression promotes proliferation, invasion, and metastasis by activating the MAPK and PI3K/AKT pathways. Consistently, knockdown of *TRIP4* increased RS in vitro and in vivo. Clinically, in a separate tissue-microarray cohort of 128 patients, high *TRIP4/hTERT* co-expression was negatively associated with OS (*p* = 0.038) [78]. Hamid et al. showed in vitro that dual inhibition of PI3K/mTOR by NVP-BEZ235 resulted in greater cytotoxicity than irradiation alone, suggesting that combined targeting of PI3K/mTOR may enhance RS [77]. Oike et al. identified a combined *KRAS/SMAD4* mutation signature exclusively in a locally relapsed tumor and in none of the 16 non-recurrent tumors in a prospective cohort of 18 patients with FIGO stage IB CC. On the other hand, 16% of patients with non-relapsed tumors harbored a *PIK3CA* mutation. Mechanistically, since no *KRASmt*/*SMAD4*mt cervical cancer cell line exists, the functional consequences were modeled in isogenic SW48 colorectal adenocarcinoma cells: combined *KRAS*^G12D activation and siRNA-mediated *SMAD4* suppression conferred significant X-ray resistance [75]. Additionally, exploratory genomic profiling in the ProfiLER database revealed *PIK3CA* mutations in about 22% of cervical squamous CC patients, indicating that the activation of the PIK3 pathways could be a factor for intrinsic RR in a subset of patients. However, no correlation with local relapse or OS was observed due to the inclusion of a relatively low number of patients [76]. Additional studies showed that targeting downstream effectors such as *IRAK1*, CD146, *TIAM2S*, and *MTA1*-containing apoptotic extracellular vesicles can increase radiosensitivity, underscoring the contribution of NF-κB, adhesion/angiogenesis and dormancy pathways to radioresistance [79,82,83,86]. Chen et al. evaluated *IRAK1* (Interleukin-1 Receptor Associated Kinase 1) in a prospective cohort of 67 patients with CC and in vitro in HeLa/SiHa models, demonstrating that high *IRAK1* mRNA expression was significantly higher in tumors from poor radiotherapy responders than in good responders (*p* = 0.0137), whereas in vitro exposure to irradiation reduced *IRAK1* levels. Furthermore, *IRAK1* knockdown inhibited NF-κB (Nuclear Factor kappa light chain enhancer of activated B cells) signaling and enhanced the anti-tumor effects of RT, while *IRAK1* overexpression activated NF-κB and reduced RT induced suppression of malignant behavior. Pharmacological NF-κB inhibition reversed the *IRAK1*-driven resistance, supporting *IRAK1*/NF-κB as a potential target to improve RS [79]. Cheng examined CD146, an adhesion molecule involved in tumor angiogenesis, and its relation with CC RS. In tissue from 50 squamous CC patients, CD146 protein was significantly overexpressed compared to normal tissue (*p* < 0.001) and was even higher in tumors classified as low radiosensitivity than in high-radiosensitivity tumors (*p* < 0.01). Functionally, blocking CD146 with antibody AA98 significantly increased radiation-induced cell death in SiHa cells, supporting CD146 as a potential target to improve RS [82]. Chuang et al. demonstrated that the short isoform *TIAM2S* (TIAM Rac1-associated GEF 2) is markedly upregulated in RR CC lines and functions as a driver of RR by reducing apoptosis, promoting proliferation and migration, and increasing lung colonization. On the other hand, *TIAMS2S* knockdown restored RS and limited metastatic potential in xenografts [83,87]. Li et al. showed that irradiation upregulates the deubiquitinase *USP21* (Ubiquitin-Specific Peptidase 21) in cervical cancer cells and high *USP21* expression correlates with radioresistance. Moreover, *USP21* stabilizes *FOXM1*, suppressing Hippo signaling through *YAP1* nuclear translocation, while *USP21* knockdown activates Hippo signaling, increases apoptosis, and significantly increases radiosensitivity in vitro and in vivo xenograft models [80,88]. Deng et al. demonstrated that irradiation-surviving tumor cells are able to secrete *MTA1* (metastasis-associated 1) in extracellular vesicles from apoptotic cells, thus inducing a dormant state via the p-*STAT1/NR2F1* (Signal Transducer and Activator of Transcription 1)/(Nuclear Receptor subfamily 2, group F, member 1) signaling axis in recipient cells. In in vitro and xenograft models, apoEV-*MTA1*–treated cells displayed reduced proliferation but enhanced survival after irradiation, linking therapy-induced dormancy to subsequent radioresistance in CC. In archived tissue from 20 patients (10 radiosensitive and 10 radioresistant), *MTA1* and caspase-3 were significantly higher in radioresistant tumors (*p* < 0.05) and strongly correlated (r = 0.6870, *p* < 0.001), while TCGA data linked high *MTA1* to reduced overall survival (log-rank * *p* = 0.0319) [86,89]. Finally, exosome-mediated MAPK/ERK activation and autophagy-related p62 signaling emerged as context-dependent modulators, with irradiation-induced exosomes generally conferring resistance, while high p62 expression in HPV-positive tumors was associated with enhanced radiosensitivity. Yoshida et al. reported that *SQSTM1*/p62 Sequestosome 1 (p62) is highly expressed in HPV-positive cervical squamous cell carcinoma at both cell line and tissue levels (seven SCC versus seven adenocarcinoma specimens); however, the expression was low or undetectable in HPV-negative adenocarcinomas. HPV-positive cell lines showed greater intrinsic RS in clonogenic assays, and siRNA-mediated p62 knockdown attenuated radiation-induced growth suppression. Notably, blocking autophagic flux without altering p62 had no effect on RS, whereas pharmacologic p62 accumulation enhanced radiation cytotoxicity, indicating an autophagy-independent mechanism. These findings suggest p62 as a candidate predictive biomarker and radiosensitization target specifically in HPV-positive cervical cancer [90]. Dong et al. demonstrated that irradiation increases the release of exosomes from HeLa cells and that exosomes derived from 4 Gy-irradiated cultures enhanced proliferation and radioresistance in both HeLa and MDA-MB-231 cells. Furthermore, irradiated cell-derived exosomes were found to upregulate p-Erk in recipient cells, indicating that the MAPK/Erk pathway mediates exosome-driven survival and radioresistance behavior after radiation exposure [91].

**Table 5:** Summary of signaling pathways, exosomes, and other mechanisms biomarkers.

First Author (Year)	Biomarker	Biological Mechanism	Type of Evidence	Study Type/Sample	Effect Direction	Conclusion
Zhou S et al. (2022) [85]	*KLK5*	Protease activity linked to carcinogenesis	Clinical In vitro In vivo	Retrospective, n = 19; HeLa *, SiHa *, ME180 *, MS751 *, and CaSki * + siRNA; murine models	↑ RR	*KLK5* overexpression correlated with radiation-resistant tumors; inhibited radiation-induced apoptosis and G2/M arrest
Song Q et al. (2022) [81]	HSP90	ATP-dependent molecular chaperone/cellular stress	In vitro In vivo	CaSki-RR * and SiHa-RR *; murine models	↑ RR	HSP90 inhibition → CD147 degradation → RS
Teng Y et al. (2025) [84]	*MICAL2*	EMT/MAPK/PI3K-AKT	Clinical In vitro In vivo	Prospective, n = 62; SiHa and HeLa *; BALB/c nude	↑ RR	*MICAL2* overexpression → ↓ RS; *MICAL2* knockdown → ↑ RS in vivo
Hernowo BS et al. (2016) [74]	*EGFR*	*EGFR* signaling/proliferation	Clinical	Retrospective, n = 32	↑ RR	*EGFR* is associated with poor RT response
Chen W et al. (2024) [79]	*IRAK1*	Innate immunity/NF-kB	Clinical In vitro In vivo	Prospective n = 67; HeLa * and SiHa * + shRNA; murine models	↑ RR	*IRAK1* KD → ↓ NF-κB → ↑ RT effect
Rowinski E et al. (2021) [76]	*PIK3CA*	PI3K/AKT/metabolism	Clinical	Prospective, N = 15	N/A	*PIK3CA* mutation in 22.3% SCC; further validation needed
Li Z et al. (2022) [80]	*USP21/FOXM1*	Cell cycle/Hippo-YAP1	In vitro In vivo	SiHa *, HeLa *, C33A *, and ME-180 *; murine models	↑ RR	*USP21* knockdown increased radiosensitivity via Hippo activation
Oike T et al. (2022) [75]	*KRAS/SMAD4* mutations	RAS/SMAD signaling	Clinical In vitro In silico	N = 18; SW48 * and SW48 * *KRAS* + siRNA	↑ RR	KRAS/SMAD4 mutations were linked to X-ray resistance
Chuang PC et al. (2025) [83]	*TIAM2S*	Cell migration/metastasis	In vitro In vivo	HeLa *, CaSki *, and C33A * + siRNA; murine models	↑ RR	TIAM2S knockdown increased radiosensitivity and reduced lung metastatic locomotion in vivo
Cheng H et al. (2016) [82]	CD146	Adhesion/angiogenesis	Clinical In vitro	Prospective, n = 50; SiHa *	↑ RR	CD146 was increased in incomplete RT response; anti-CD146 increased radiosensitivity
Hamid MB et al. (2021) [77]	*EGFR*/PI3K/mTOR/Bcl-2	*EGFR* + PI3K/mTOR signaling	In vitro	HeLa *	↑ RR	Dual PI3K-mTOR targeting may radiosensitize cells
Che Y et al. (2019) [78]	*TRIP4/hTERT*	MAPK/PI3K-AKT/telomerase	Clinical In vitro In vivo	Prospective, n = 128 patients; HeLa *, SiHa *, C33A *, DoTc2 *, HeLa S3 *, Caski *, and Ect1 * + siRNA; murine	↑ RR	*TRIP4* promoted radioresistance via hTERT upregulation
Deng YR et al. (2025) [86]	*MTA1* via apoEVs	Dormancy/extracellular vesicles	Clinical In vitro In vivo	Retrospective, n = 20 (FFPE CC tissues) n = 6 (FS); HeLa * and SiHa *; murine models	↑ RR	ApoEV-*MTA1* promotes cellular dormancy via the p-*STAT1/NR2F1* signaling axis
Yoshida M et al. (2025) [90]	*SQSTM1*/p62	Autophagy independent, HPV-dependent	Clinical In vitro	n = 14; C33A *, HeLa *, ME180 * SiHa *, CaSki *, and SKG IIIa * + siRNA	↑ RS (HPV+)	p62 enhances HPV-related radiosensitivity
Dong Y et al. (2024) [91]	Exosomes/MAPK-ERK	Exosome-mediated signaling	In vitro	HeLa * and MDA-MB-231 *	↑ RR	Irradiation-induced exosomes promote radioresistance via MAPK/ERK signaling

Abbreviations: *, cell lines (human cell models); CC, cervical cancer; FFPE, formalin-fixed, paraffin-embedded; FS, fresh samples; RR, radioresistance; RS, radiosensitivity; RT, radiotherapy.

## 4. Discussion

**Figure 2:**
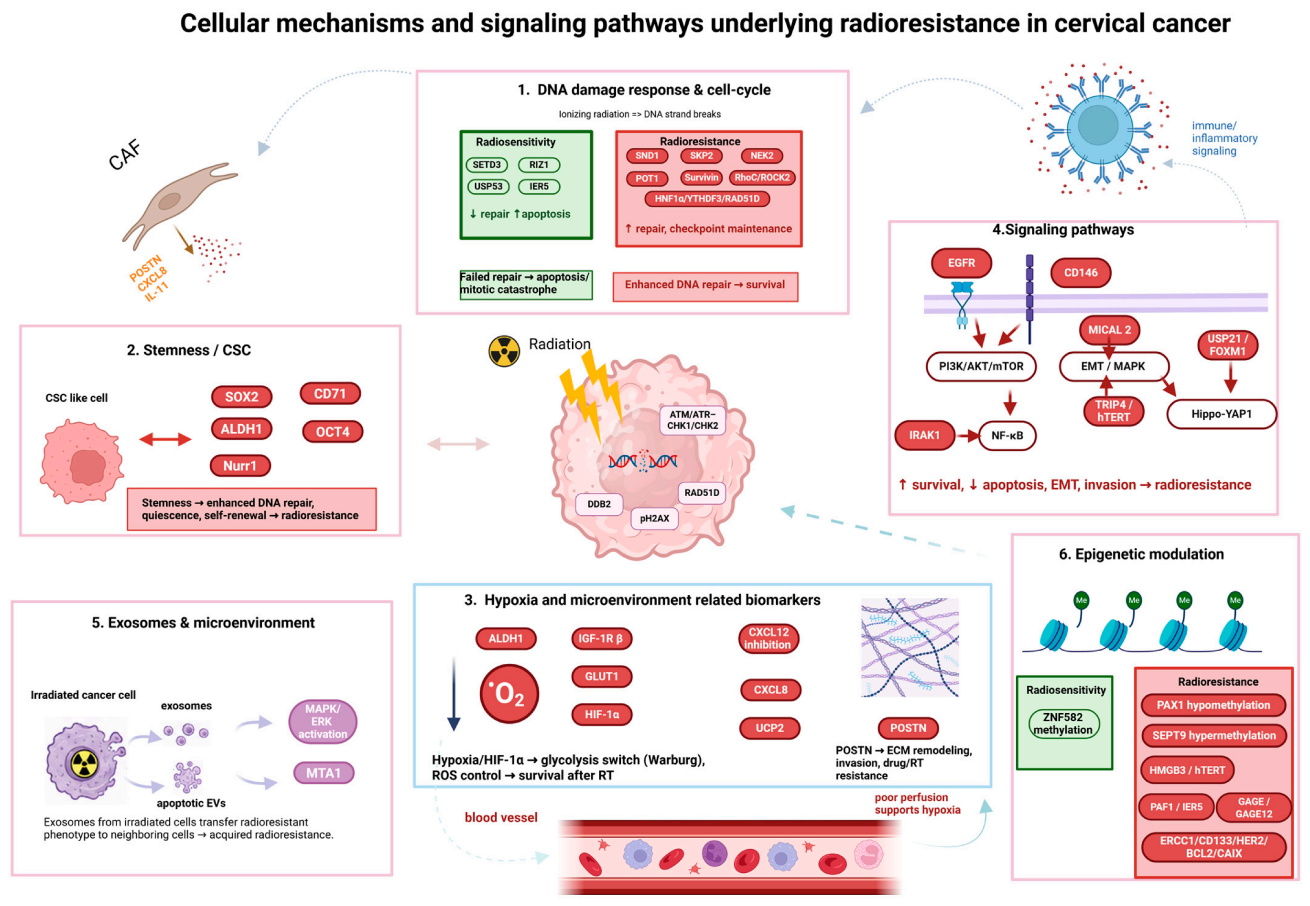
Cellular mechanisms and signaling pathways underlying radioresistance in cervical cancer. Created in BioRender. Girbovan, A. (2026) https://BioRender.com/uiqz6x2 (accessed on 6 June 2026).

The review emphasizes that radiosensitivity in cervical cancer is controlled by a complex but convergent network of molecular mechanisms rather than individual biomarkers. Although the included studies identified a broad range of candidate biomarkers, most clustered into a limited number of biological processes, including DNA damage response and cell cycle regulation, cancer stemness, hypoxia and microenvironment-related adaptation, epigenetic and transcriptional regulation, exosomes, and signaling pathway activation (Figure 2). Furthermore, the majority of biomarkers identified in the reviewed studies were related to radioresistance rather than radiosensitivity, suggesting that treatment failure in cervical cancer is more often due to activation of certain resistance pathways rather than a passive failure to respond to irradiation.

A schematic representation of key cellular processes implicated in cervical cancer radioresistance and radiosensitivity, including enhanced DNA damage response and cell cycle control, enrichment of cancer stem-like cells, hypoxia and microenvironment reprogramming, signaling pathways, epigenetic regulation, and exosome-mediated intercellular communication, is shown in Figure 2. Biomarkers associated with radiosensitization (e.g., *SETD3*, *RIZ1*, *IER5*, *USP53*, and *ZNF582* methylation) and radioresistance (e.g., *SKP2*, *NEK2*, *SOX2*, *ALDH1*, *IGF-1Rβ*, *GLUT1*, and *POSTN*) are indicated within each module.

Biomarkers related to DNA damage response and cell cycle control accounted for one-third of the studies included in this systematic review. Several molecules, including *SKP2*, *NEK2*, *HNF1α/YTHDF3/RAD51D*, *RhoC/ROCK2*, *SND1*, and *POT1*, promoted radioresistance by enhancing DNA repair, maintaining checkpoint control, or preserving genomic stability after irradiation. Among these, only *NEK2* and the *HNF1α/YTHDF3/RAD51D* axis were validated in vitro, in vivo, and in clinical cohorts [39,40]. Concerning radiosensitivity, several biomarkers were identified: *SETD3*, *RIZ1*, *USP53*, and, to some extent, *IER5*, by promoting apoptosis, impairment of repair capacity, or disruption of cell cycle progression [27,30,32,35]. *SETD3* was the only biomarker validated in vitro, in vivo, and clinically. Several potential therapeutic targets were identified, including *SETD3*, *RIZ1*, and *USP53* as candidate radiosensitizing factors, whereas *NEK2*, survivin, *POT1*, the *HNF1α/YTHDF3/RAD51D* axis, and the *RhoC/ROCK2* pathway appear to promote radioresistance and may be amenable to pharmacologic blockade [41,44,46]. These findings support the idea that the balance between efficient repair of irradiation-induced damage and irreversible entry into apoptosis or mitotic catastrophe is a major determinant of radiotherapy response in CC.

Another mechanism of radioresistance identified was the contribution of stemness-associated phenotypes and tumor plasticity. *SOX2* was the most consistently validated stemness-related marker and was repeatedly associated with poorer clinical outcomes, including local recurrence, shorter survival, and reduced disease-free intervals. However, divergent information regarding squamous histologies emerged in head and neck cancers and lung cancer, where *SOX2* high groups were correlated with better survival and better response to radiotherapy [92,93]. ALDH1, *Nurr1*, and CD71 further supported the hypothesis that cervical tumors enriched for stem cell-like features are more prone to survive irradiation and repopulate following treatment. Similar findings regarding ALDH1 and its role in radioresistance have been reported in other oncological pathologies such as head and neck squamous cancers and breast cancer [94,95,96]. Inhibitors of ALDH1, such as disulfiram combined with copper, have been shown to enhance radiosensitivity in vitro and in vivo in chondrosarcoma, suggesting that this strategy could also represent a potential therapeutic approach in cervical cancer [97]. These observations are biologically reasonable in view of the increased self-renewal, improved oxidative stress tolerance, and superior DNA repair capacity of cancer stem-like cells [98]. In this context, the interaction between HPV oncogenic signaling and the acquisition of a stem-like phenotype is particularly notable, especially for biomarkers such as *Nurr1* and CD71, which may identify a subgroup of HPV-driven tumors with distinct radiobiological behavior [52,54].

Hypoxia and microenvironment-related biomarkers also emerged as a recurrent mechanism of resistance, although the number of clinically validated studies in this category was smaller. Among the biomarkers clinically validated were IGF-1Rβ, GLUT1, *POSTN*, and *CXCL8* [56,58,60]. High IGF-1Rβ expression and, to a lesser extent, GLUT1 overexpression were associated with poor OS in locally advanced disease [56]. These findings are consistent with data from head and neck squamous cell carcinomas, where IGF-1R overexpression associates with radiotherapy failure and poorer survival; IGF-1Rβ may similarly contribute to radioresistance in cervical cancer and represents a potential therapeutic target [99,100]. *POSTN* was associated not just with incomplete response and inferior OS: an exploratory in silico analysis also suggested a potential link with resistance to several chemotherapeutic and targeted agents [60]. As with several other candidates, the clinical evidence for *CXCL8* is currently limited to differential expression between tumor and normal tissues, and its functional role in radioresistance has been established only in vitro (HeLa-RR and SiHa-RR models); therefore, studies correlating *CXCL8* with radiotherapy outcomes in patient cohorts are still needed. Other markers validated only in vitro and/or in vivo were ALDH1, *CXCL12*, and *UCP2*, which contribute to an adaptive microenvironment that promotes survival after irradiation through metabolic reprogramming, oxidative stress control, extracellular signaling, and stemness maintenance [55,57,61]. These findings suggest that radioresistance is not exclusively intrinsic but is also shaped by tumor–stroma interactions, inflammatory signaling, and hypoxia.

Another important finding of this review is that epigenetic and transcriptional regulation seems to be a higher-order layer integrating multiple resistance pathways. Several candidates, including *SEPT9* and *PAF1*, remain supported almost exclusively by in vitro and in vivo evidence, with their link to RR inferred from tumor versus normal expression rather than from radiotherapy outcomes. Further studies are required for clinical validation. On the other hand, *PAX1*, *ZNF582*, *GAGE*, *HMGB3/hTERT*, and a multi-marker proteomic panel have been correlated with genuine radiotherapy endpoints (Table 6). *PAX1* hypomethylation and *GAGE12*-mediated chromatin remodeling were associated with poor radiotherapy response [65,67,69]. Higher *ZNF582* promoter methylation correlated with improved radiosensitivity, while overexpression of *ZNF582* promoted radioresistance [66]. These findings suggest that not all methylation changes are biologically equal. The functional effect depends on the gene and on whether methylation suppresses a radioresistance pathway or a radiosensitizing one. For *ZNF582*, promoter methylation reduces the expression of a protein associated with radioresistance, so higher methylation levels are linked to increased radiosensitivity. Moreover, *ZNF582* has also been identified as a potential biomarker for cervical cancer screening, highlighting its broader clinical relevance [101]. Concerning *PAX1*, the clinical evidence rests on a single-institution study with a small hypomethylated subgroup (n = 15, 12%) correlated with RT response at 3 months (multivariate OR 4.433, 95% CI 1.131–17.380, * *p* = 0.033), resulting in a wide confidence interval and limited to assessing short-term radiotherapy response, without data on OS or recurrence outcomes [65]. Furthermore, *HMGB3/hTERT* and *PAF1/IER5* illustrate how transcriptional regulation can influence both telomere-associated survival and immediate early stress responses [29,70]. The RPPA-derived five-marker panel and the subsequent radiosensitivity score further indicate that multi-marker approaches may capture more accurately the biological complexity of treatment response than any single biomarker alone, although validation outside the derivation cohort remains limited [73].

The signaling pathway section of the review further supports that apparently different biomarkers converge on a small number of shared survival pathways. Canonical oncogenic pathways included *EGFR*, *KRAS/SMAD4*, *PIK3CA*, mTOR/Bcl-2, and *TRIP4/hTERT* [74,75,76,77,78]. The evidence for the *KRASmt/SMAD4*mt signature remains preliminary as it was detected in only a single patient, and its functional validation relied on a non-cervical (colorectal) cell model, so this study contributes mechanistic rather than clinical-endpoint evidence [75]. Non-canonical mediators such as *IRAK1*, *USP21/FOXM1*, HSP90, CD146, *TIAM2S*, *MICAL2*, and *KLK5* revealed NF-κB activation, Hippo/YAP dysregulation, adhesion signaling, epithelial–mesenchymal transition, and metastatic behavior ]. In addition, the presence of exosome-mediated transfer of resistance signals, in particular by irradiated-cell exosomes and *MTA1*-containing apoptotic extracellular vesicles, suggests that radioresistance may be actively propagated between tumor cells via microenvironmental communication and dormancy induction [86]. These findings are consistent with the literature, especially in nasopharyngeal cancers where exosome signaling with MTA-1-associated pathways augments invasiveness and therapy resistance [102,103].

From a clinical perspective, the biomarkers with the most robust validation in this review were those associated with endpoints such as overall survival, disease-free survival, local recurrence, or residual disease after treatment. These include *SKP2, SOX2*, ALDH1, IGF-1Rβ, *GLUT1*, *POSTN*, *EGFR*, *PAX1*, *ZNF582*, *SEPT9*, and the RPPA-based panel [36,47,48,51,56,60,65,66,67,73,74]. However, even for these markers, the level of evidence remains unbalanced, as many studies have included small cohorts, single-institution populations, and variable endpoint definitions. Until prospective, multicenter validation studies with standardized assays and pre-specified endpoints are available, these biomarkers should be regarded only as exploratory rather than practice changing. In addition, several highly promising biomarkers such as HSP90, CD44+, *USP21*, *MICAL2*, and *TIAM2S* are currently supported mainly by pre-clinical data and therefore require prospective external validation before they can be considered for routine use [57,80,81,83].

In addition to the biological characterization of radiotherapy biomarkers for assessing tumor response, an equally important but less explored aspect is the available technologies for detection and dynamic monitoring [104]. To overcome these limitations, electrochemical biosensors were designed that are characterized by cost effectiveness, fast reaction time, ease of use, miniaturization possibilities, and high sensitivity and specificity [105]. Multiple electrochemical biosensors in CC have been developed for several biomarkers such as p16INK4A, p53, Ki67, HPV-16, HPV-18, and squamous cell carcinoma antigen (SCCA) [106,107,108,109,110,111,112,113]. Theoretically, such dynamic monitoring using different biomarkers in radioresistance could enable early identification of radioresistant tumors and could further lead to adaptation of early evaluation and treatment strategies [114]. Integrating highly sensitive electrochemical platforms with validated biomarkers represents a compelling direction for future translational research [115].

Several methodological limitations should also be acknowledged. Firstly, the included articles were highly heterogeneous with respect to study design and radiation schedules used in vitro and in vivo. Secondly, many studies relied on a limited set of cervical cancer cell lines, particularly HeLa, which is derived from adenocarcinoma and therefore does not reflect the most common squamous histological subtype of cervical cancer. Another cell line frequently used along HeLa was SiHa, a derived squamous cell carcinoma with an HPV-16-integrated genome; however, it may not fully represent the biological diversity of cervical cancer, particularly across HPV status. Thirdly, the current evidence has not been externally validated, as most of the biomarkers have been evaluated within single institution cohorts, without replication in independent datasets. However, *POSTN* was evaluated on a TCGA cohort in addition to a local cohort [60]. A notable exception is *ZNF582* promoter methylation, whose predictive value for radiotherapy response was confirmed in a second independent multicenter cohort recruited from three centers, thus providing external replication [66]. Furthermore, a 7-factor multivariate Cox model based on a reverse-phase protein assay was externally validated on a TCGA cohort. On the other hand, few studies have assessed the incremental predictive values of the biomarkers over established clinical parameters such as FIGO stage and lymph node status and whether their addition would add clinical risk stratification above standard clinicopathological factors. Future research should therefore prioritize the development of standardized detection assays, the integration of multi-marker panels, and the conduct of prospective, adequately powered, multicenter validation studies. The decision to exclude non-coding RNAs and exogenous radiosensitizers was made to narrow the scope of this review to endogenous molecular biomarkers. However, this further implies that the present review represents only one part of the broader radiobiological landscape.

Finally, some limitations relate to the review methodology itself. The search was limited to PubMed and databases such as Scopus, Web of Science, and Embase, which may have resulted in the omission of relevant studies. Therefore, publication or selection bias cannot be fully excluded, which should be considered when weighing the strength of the conclusions. In addition, the review was not prospectively registered a priori, limiting the transparency of the protocol.

**Table 6:** Summary clinically validated biomarkers associated with radiotherapy outcomes.

First Author (Year)	Biomarker	Mechanism Category	Effect Direction	Detection Method	Clinical Endpoint	Study Design	No of Pts	Key Limitations
Li Q et al. (2019) [30]	*SETD3*	DDR and cell cycle checkpoints	↑ RS	RT-qPCR	↑ RT response (Kitahara et al.) [116] *p* < 0.01	Clinical prospective in vitro + in vivo	9	Small cohort, RT response classification only, no survival or recurrence data
Du H et al. (2023) [40]	*HNF1α/YTHDF3/RAD51D*	DDR and cell cycle checkpoints	↑ RR	RT-qPCR	↓ RT response (Ke et al.) [117] *p* < 0.01	Clinical prospective in vitro + in vivo	20	Small cohort, RT response classification only, no survival or recurrence data
Zhou Q et al. (2020) [35]	*USP53*	DDR and cell cycle checkpoints	↑ RR	IHC	↓ RT response (CR/PR/SD) *p* = 0.048	Clinical retrospective in vitro		Small cohort, retrospective, RT response classification only, mostly FIGO stage II; survival data collected, but RT response is the endpoint reported
Fu HC et al. (2017) [36]	*SKP2*	DDR and cell cycle checkpoints	↑ RR	IHC	↑ LRR HR 3.76 *p* < 0.001 ↓ DFS *p* < 0.001 ↓ OS *p* < 0.001	Clinical retrospective	149	Retrospective design, treatment era 2004–2006, non-standardized treatment: only 97/149 (65%) received RT + BT
Huang C et al. (2018) [47]	*SOX2*	cancer stem cell/stemness	↑ RR	IHC	↓ OS *p* < 0.01 ↓ LR *p* = 0.05	Clinical prospective in vitro + in vivo	56	Small cohort
Fahmi MN et al. (2023) [47,51]	ALDH1	cancer stem cell/stemness	↑ RR	IHC	No-CR ALDH ≥ 166.05 → OR 3.127 *p* = 0.043	Clinical retrospective	58	Small cohort, retrospective design RT response only, No OS, LR data modest AUC (0.682)
Moreno-Acosta P et al. (2017) [56]	IGF-1Rβ GLUT1	hypoxia and microenvironment	↑ RR	IHC	IGF-1Rβ ↓ OS *p* = 0.007 GLUT1 ↓ OS *p* = 0.05	Clinical prospective	149	*GLUT1* only marginal effect on OS, needs confirmation in larger cohorts
Huang CQ et al. (2025) [60]	*POSTN*	hypoxia and microenvironment	↑RR	IHC (n = 153) + mRNA/TCGA (n = 92)	↓ RT response TCGA *p* = 0.034 Clinical cohort *p* = 0.028 OS TCGA HR 2.721, *p* = 0.011 Clinical cohort HR 4.005, *p* < 0.001	Clinical retrospective + bioinformatic (TCGA) in vitro	92 + 153	Retrospective cohort validation, mechanistic validation limited to a single cell line
Li X et al. (2023) [65]	*PAX1* hypo-methylation	epigenetic and transcriptional regulation biomarkers	↑ RR	QMSP	↓ RT response *p* = 0.033	Clinical prospective in vitro	125	Small hypomethylated subgroup (n = 15), wide CI RT response only, no OS, LR data
Li Z et al. (2020) [70]	*HMGB3/hTERT*	epigenetic and transcriptional regulation biomarkers	↑ RR	IHC	↓ RT response *HMGB3* *p* = 0.040 hTERT *p* = 0.049	Clinical prospective in vitro + in vivo	53	RT response only, no OS, LR data
Wu N et al. (2022) [66]	*ZNF582* methylation	epigenetic and transcriptional regulation biomarkers	↑ RS (methylation)/↑ RR (protein)	qPCR	↑ RT response *p* < 0.001	Clinical prospective multicenter in vitro + in vivo	50 + 50	RT response only, no OS, LR data
Choi CH et al. (2016) [72]	*ERCC1*/CD133/HER2/BCL2/CAIX	epigenetic and transcriptional regulation biomarkers	↑ RR (panel high)	RPPA (validated by WB + IHC)	↓ PFS *p* < 0.001 ↓ OS *p* < 0.001	Clinical–prospective; internal (train/test) + external (TCGA) validation	181	External TCGA validation used RNA (not protein) and did not clearly reproduce significance
Nin DS et al. (2021) [69]	*GAGE/GAGE12*	epigenetic and transcriptional regulation biomarkers	↑ RR	IHC, qRT-PCR (pre-treatment biopsy)	Recurrence/metastasis at 2 y ↑ *GAGE*—mRNA *p* = 0.0028; IHC intensity *p* = 0.026	Clinical retrospective (archival FFPE) in vitro + in vivo	43	Small cohort, retrospective design, protein H-score not statistically significant (*p* = 0.064)
Zhou S et al. (2022) [85]	*KLK5*	signaling pathways, exosomes, and other mechanisms	↑ RR	Microarray RT-qPCR WB	Recurrence/metastasis at 3 y	Clinical retrospective in vitro + in vivo	19	Very small cohort, no clinical *p*-value/multivariate analysis
Teng Y et al. (2025) [84]	*MICAL2*	signaling pathways, exosomes, and other mechanisms	↑ RR	WB IHC	↓ RT response *p* = 0.006 PFS *p* = 0.032	Clinical prospective in vitro + in vivo	62	Small cohort; OS not significant (limited follow-up)
Hernowo BS et al. (2016) [74]	*EGFR*	signaling pathways, exosomes, and other mechanisms	↑ RR	IHC	↓ RT response *p* = 0.0001	Clinical retrospective	32	Small cohort; adenocarcinoma only; RT response only, no OS, LR data
Chen W et al. (2024) [79]	*IRAK1*	signaling pathways, exosomes, and other mechanisms	↑ RR	RT-qPCR	↓ RT response *p* = 0.0137	Clinical prospective in vitro + in vivo	68	Small cohort; RT response only, no OS, LR data

Abbreviations: BT, brachytherapy; CR, complete response; DDR, DNA damage response; FIGO, Federation International of Gynecology and Obstetrics; IHC, immunohistochemistry; LLR, Locoregional recurrence; LR, local recurrence; PR, partial response; PFS, progression-free survival; RT-qPCR, reverse transcription quantitative polymerase chain reaction; RPPA, reverse-phase protein array; RS, radiosensitivity; RR, radioresistance; RT, radiotherapy; SD, stable disease; OS, overall survival; QMSP, quantitative methylation-specific polymerase chain reaction; qPCR, quantitative polymerase chain reaction; WB, Western blot.

The evidence summarized in this systematic review suggests that the most informative model of cervical cancer radioresistance is not based on single genes but on interconnected biological pathways. Future studies should therefore prioritize integrated biomarker strategies that combine genomic, epigenetic, and proteomic pathways, ideally in prospective cohorts with standardized clinical endpoints. Distinct attention should be given to biomarkers that reflect convergent mechanisms such as DDR proficiency, stemness, hypoxia adaptation, and activation of various pathways such as MAPK/PI3K-AKT and Hippo/YAP. This approach may improve the ability to identify patients at risk of radioresistance, with possible residual disease after treatment, enabling personalized treatment intensification or biomarker-guided radiosensitization in cervical cancer.

## 5. Conclusions

This systematic review shows that multiple pathways are implicated in radioresistance, including DNA damage response and cell cycle regulation, cancer stemness, hypoxia and metabolic adaptation, epigenetic modulation, and aberrant activation of signaling pathways, all of which contribute to treatment failure. Among the investigated markers, those with the most robust clinical associations include *SKP2*, *SOX2*, ALDH1, P16INK4A, IGF-1Rβ, *GLUT1*, *POSTN*, *EGFR* and epigenetic regulators such as *PAX1* and *ZNF582*. Further studies should focus on integrated biomarker strategies in well-designed prospective cohorts, emphasizing markers of DDR proficiency, stemness, hypoxia adaptation, and key signaling pathways. This could improve the identification of radioresistant patients and improve outcomes for patients with cervical cancer.

## Data Availability

No new data were created or analyzed in this study. Data sharing is not applicable to this article.
